# Stem Cell Therapy in Lupus

**DOI:** 10.2478/rir-2022-0011

**Published:** 2022-07-06

**Authors:** Xinran Yuan, Lingyun Sun

**Affiliations:** 1 Department of Rheumatology and Immunology, The Affiliated Drum Tower Hospital of Nanjing University Medical School, Nanjing, Jiangsu Province, China

**Keywords:** systemic lupus erythematosus, stem cell therapy, hematopoietic stem cells, mesenchymal stem cells, transplantation

## Abstract

Systemic lupus erythematosus (SLE) is a chronic autoimmune and inflammatory disease with multiple organs and systems involved such as the kidney, lung, brain and the hematopoietic system. Although increased knowledge of the disease pathogenesis has improved treatment options, current immunosuppressive therapies have failed to prevent disease relapse in more than half of treated patients. Thus, the cell replacement therapy approach that aims to overcome adverse events of traditional treatment and improve recovery rate of refractory SLE is considered as an alternative treatment option. A large number of animal studies and clinical trials have shown stem cell therapy to be a promising therapeutic approach for the treatment of SLE. Since the first transplantation into human patients, several stem cell types have been applied in this field, including hematopoietic stem cells (HSCs) and mesenchymal stem cells (MSCs). In this review, we overview different cell sources of stem cells and applications of the stem cell therapy for treatment of SLE, as well as the comparison between HSCs transplantation (HSCT) and MSCs transplantation (MSCT).

## Introduction

Systemic lupus erythematosus (SLE) is a systemic autoimmune disease (AD) with diverse clinical symptoms due to multiple organ injury, such as the kidney, lung, brain, and the hematopoietic system, leading to a high morbidity and mortality. Corticosteroids and cyclophosphamide (CTX) and mycophenolate mofetil (MMF) are the most classically and widely administered medications, which have led to a significant improvement in survival over the last few decades and decreased the progression to end-stage multi-organ failure. In addition to conventional immunosuppressive therapies, several new strategies have been developed to target specific activation pathways relevant to SLE pathogenesis,^[1]^ such as rituximab (B-cell depleting therapy), epratuzumab (B-cell modulating therapy), belimumab (inhibition of B-cell survival), abatacept and toralizumab (inhibition of T-cell function), tocilizumab (IL-6 inhibition), and sifalimumab and rontalizumab (type I interferon inhibitors).^[2,3,4]^ Although these drugs have led to a markedly improved outcome in SLE, disease control remains unsatisfactory in a subset of patients. Moreover, use of these agents often leads to serious adverse effects or relapses after discontinuation. Therefore, it is of great clinical significance to explore new treatment methods other than conventional drugs, such as stem cell transplantation, for patients with refractory SLE.

Since being discovered over half a century ago, stem cells have been investigated extensively to characterize their cellular and physiological influences. Stem cells were found to modulate immune responses by regulating the function and fate of cells in the innate and adaptive immune system.^[5,6]^ Their properties make stem cells a promising therapeutic paradigm for ADs. This review will focus on the current progress and established evidence of stem cell therapy for SLE patients.

### Hematopoietic Stem Cells (HSCs)

HSCs were first discovered in 1961,^[7]^ and are the stem cells that give rise to other blood cells.^[8]^ HSCs transplant (HSCT) was first used in AD in 1997: the patient with hematologic malignancies and autoimmune conditions receives HSCT, and in long-term follow-up, the malignant and autoimmune conditions have clinical improvement.^[9]^ During the same year, Marmont *et al*.^[10]^ reported a successful application of HSCT for a 46-year-old SLE patient. Since then, HSCT has been used to treat a variety of ADs, such as SLE, rheumatoid arthritis (RA), multiple sclerosis (MS), and systemic sclerosis (SSc).^[11]^ A large number of clinical studies of autologous HSCT in the treatment of SLE have been carried out worldwide and gratifying clinical results have been achieved, which provides the possibility for the fundamental cure of SLE.

## HSC Deficiency in SLE

The lupus conditions might impair the functional integrity of HSCs. Westerweel *et al*.^[12]^ reported that SLE patients had lower levels of circulating HSCs and endothelial progenitor cells (EPCs), and the depletion of HSCs was associated with the increased apoptosis of HSCs. The progenitor cell–mediated endogenous vascular repair was defective, and thus SLE patients had higher prevalence of atherosclerosis. Pyrovolaki *et al*.^[13]^ found the proportion of CD40^+^ cells within the CD34^+^ fraction was increased as compared with controls, which contributed to the amplification of Fas-mediated apoptosis of HSCs. Zheng *et al*.^[14]^ proposed that there was a link between ADs and defects in HSCs. They found that due to hyperactivation of mTOR (mammalian target of rapamycin), the HSCs in the Scurfy mice with Foxp3 mutation were extremely poor in hematopoiesis. However, Niu *et al*.^[15]^ reported that the HSCs were significantly expanded in lupus mice and the inflammatory conditions of lupus led to HSC mobilization and lineage-biased hematopoiesis. The discrepancy might be associated with the stage of HSCs or the method of animal experiment, which need further investigation.

## HSCT in SLE

### Prospective Studies

The prospective study of HSCT in SLE has been limited. In 1998, Burt *et al*.^[16]^ first reported 10 patients with these ADs (2 SLE, 2 RA, and 6 MS) who received HSCT. The HSCs were collected from bone marrow or mobilized with either granulocyte colony-stimulating factor (G-CSF) or CTX and G-CSF. The stem cells were enriched and reinfused after immunosuppression with 200 mg/kg CTX, 4 g methylprednisolone, and 90 mg/kg antithymocyte globulin (ATG). The authors found that T-cell–depleted HSCT was safely used to treat these 10 patients with severe AD, and all patients have demonstrated stabilization or improvement during follow-up (5–17 months). In 2000, Traynor *et al*.^[17]^ performed a phase I study to analyze the safety and efficacy of high-dose chemotherapy and HSCT in the treatment of severe SLE. The autologous HSCs were mobilized with 2.0 g/m^2^ CTX and 10 μg/kg G-CSF, enriched using CD34^+^ selection, and reinfused after immunosuppression with 200 mg/kg CTX, 1 g methylprednisolone, and 90 mg/kg ATG. Finally, 7 patients received high-dose chemotherapy and HSC infusion. During the follow-up (12–40 months), all patients remained free from active lupus and improved continuously after HSCT, with no immunosuppressive medication or small residual doses of prednisone. During the same year, Rosen *et al*.^[18]^ performed a phase 1/2 study in 7 patients (1 polychondritis, 3 treatment-refractory SLE, and 3 SSc). HSCs were mobilized and immunosuppressed as in the previous study. The authors suggested that in-vivo immunoablation in combination with autologous HSCT can block the autoimmune process in relapsing polychondritis or SLE without the incidence of severe infections. Burt and Traynor^[19]^ began a National Institutes of Health–funded phase III clinical trial of HSCT for refractory SLE since 2003. In 2006, Burt *et al*.^[20]^ reported a large single-arm prospective trial of autologous HSCT in refractory SLE, with 50 patients enrolled in the study. Peripheral blood stem cells were mobilized with 2.0 g/m^2^ CTX and G-CSF, enriched ex vivo by CD34^+^ immunoselection, cryopreserved, and reinfused after treatment with 200 mg/kg CTX and 90 mg/kg ATG. With a mean follow-up of 29 months (from 6 months to 7.5 years) for patients receiving HSCT, overall 5-year survival was 84% and probability of disease-free survival at 5 years after HSCT was 50%. Secondary analysis demonstrated significant improvement in systemic lupus erythematosus disease activity index (SLEDAI) score, ANA (Anti-nuclear antibody), anti-ds DNA, complement, and carbon monoxide diffusion lung capacity adjusted for hemoglobin, and the renal function kept stable. Toxicities included 2% treatment-related mortality, 2 patients requiring intubation, and infectious complications including bacteremia/endocarditis, fungemia, peritonitis, zoster, and PCP (pneumocystis carinii) pneumonia. The study confirmed that HSCT was effective in the treatment for refractory SLE. Also in 2006, Tsukamoto *et al*.^[21]^ performed a phase I–II trial of autologous peripheral blood stem cell transplantation in the treatment of refractory AD, with 8 patients enrolled (5 SSc, 1 SSC + SLE, and 1 dermatomyositis). The authors suggested that high-dose CTX (2.0 g/m^2^) with autologous HSCT was feasible and might be effective in the treatment of severe and refractory AD. In 2009, Alexander *et al*.^[22]^ reported the results of a single-center prospective study of long-term immune reconstitution after autologous HSCT in 7 patients with SLE. At the last follow-up, all 7 patients except 1 achieved long-lasting clinical and serologic remissions and were no longer reliant on immunosuppressive therapy. The one exception relapsed after having been in clinical remission for more than a year. Moreover, the authors found the clinical remissions were accompanied by the depletion of autoreactive immunologic memory. After the HSCT, the pathogenic anti–double-stranded DNA (anti-ds DNA) antibodies and protective antibodies in serum disappeared, and the adaptive immune system had a fundamental resetting.

### Retrospective Studies

Several retrospective studies also suggested that auto-HSCT might be able to achieve disease control for SLE. In 2004, a retrospective registry survey was carried out by the European Blood and Marrow Transplant and European League Against Rheumatism (EBMT/EULAR) registry.^[23,24]^ A total of 53 SLE patients from 23 centers were treated with HSCT. Remission rate of disease activity (SLEDAI < 3) was 66% during 6-months’ follow-up after HSCT, 32% of which relapsed after 6 months. The authors suggested that the relapse rate might be reduced by long-term immunosuppressive therapy post-HSCT. In 2007, Loh *et al*.^[25]^ performed a retrospective investigation of SLE patients receiving auto-HSCT to determine the prevalence of significant cardiac involvement and the impact of transplantation on it. A total of 55 patients were enrolled in the study, of which 13 were found to have abnormal cardiac findings before HSCT. There were no transplant-related or cardiac deaths during follow-up, and the patients with impaired cardiac function remained stable or improved. In recent years, several Chinese centers reported the longterm outcomes of HSCT for SLE. Cao *et al*.^[26]^ reviewed 22 SLE patients undergoing autologous HSCT. All the patients survived over 3 years, and the 3-year progression-free survival (PFS) was 77.27%. The 5-year overall survival rate and PFS were 95.20% and 67.90%. Leng *et al*.^[27]^ reported a 10-year follow-up outcome of HSCT. A total of 27 severe SLE patients received combination therapy with high-dose immunosuppressive therapy and autologous peripheral HSCT. At 6 months follow-up, 21 patients (87.5%) achieved remission, 2 partial remissions, and 1 died. At the end, 14 patients completed the 10-year follow-up. The 10-year overall survival rate and 10-year remission survival rate were both 86.0%. The median proteinuria level was significantly decreased during follow-up.

## Problems in HSCT for SLE

It has been reported that autologous HSCT is successful in treating SLE; however, there are still many problems that further need to be solved. First, the relapse after transplantation remains an insurmountable problem. In a previous study, the relapse rate is nearly one-third. In the EBMT/EULAR study, 32% of SLE patients relapsed after 6 months of receiving HSCT.^[23]^ Second, the post-transplantation complications persisted for many years. The most common short-term complication after HSCT is infection, such as CMV (cytomegalovirus) or bacterial/fungal infections. Burt *et al*.^[20]^ reported that 50 SLE patients received HSCT and the infection rate was relatively high before, during, and after transplantation. One death occurred from disseminated mucormycosis, 1 patient had pneumocystis jiroveci pneumonia on bronchoscopy and esophageal candidiasis, and 14 patients had bacteremia (predominantly gram positive) during transplantation. Positive stool culture results were seen in 4 patients: 3 for *Clostridium difficile* and 1 for *Salmonella*. Two patients developed CMV disease within 100 d after transplantation. Song *et al*.^[28]^ reported that the main adverse events after HSCT for SLE included allergy, infection, elevation of liver enzymes, bone pain, and heart failure, and 2 patients died due to severe pneumonia and heart failure. The common long-term complication after HSCT is secondary ADs, which might be associated with the imbalance between autoimmunity and tolerance during immune reconstitution following intense immunosuppression during HSCT. Daikeler *et al*.^[29]^ performed a retrospective study of the EBMT AD Working Party to specify the incidence and risk factors for secondary AD after HSCT. After autologous HSCT, 29 of 347 patients developed at least one secondary AD, and after allogeneic HSCT, 3 of 16 patients developed secondary AD, including autoimmune hemolytic anemia, acquired hemophilia, autoimmune thrombocytopenia, antiphospholipid syndrome, thyroiditis, Graves’ disease, myasthenia gravis, RA, *et al*. The authors further performed multivariate analysis and found that the risk factors for secondary AD were: (1) SLE as primary AD and (2) ATG use plus CD34^+^ graft selection. Also, the cost of HSCT is expensive, which limits its clinical application. According to the data form EBMT, the number of autoimmune patients receiving HSCT was about 200/year in 2015–2018, and mostly used in MS and SSc.^[30]^ Under the impact of COVID-19, transplant activity for AD decreased by 52% in 2020.^[31]^ In general, HSCT provides “remedial therapy” for patients who are refractory and ineffective with other therapies. However, further randomized control experiments are still needed to confirm whether it is more effective.

## Mesenchymal Stem Cells (MSCs)

### Biological Characteristics of MSCs

MSCs are considered as non-hematopoietic multipotent plastic-adherent fibroblastic cells that are found in embryos and adults; they have reparative potential through self-renewal and differentiation capacities and are recognized as being able to modulate immune responses.^[32,33]^ Under certain inducement conditions, MSCs can differentiate into connective tissue cells, such as adipocytes, osteoblasts, chondrocytes, myoblasts, and early progenitors of neural cells.^[34]^ MSCs exist in all organs including connective tissue, besides bone marrow, and they can be isolated from adult and fetal tissues containing umbilical cord (UC), cord blood, circulating blood, amniotic fluid, skeletal muscle, adipose tissue, synovial tissue, and the placenta.^[35,36]^ MSCs have the characteristics of easy purification, rapid expansion in vitro, and long-term passage. Currently, UC-MSCs have become the main cell types in MSCs treatment because of their more primitive and extensive sources.

### MSC Deficiency in SLE

There was evidence showing that MSCs deficiency was involved in SLE pathogenesis. Bone marrow derived MSCs (BM-MSCs) obtained from SLE patients exhibited impaired capacities of proliferation, differentiation, secretion of cytokines, and immune modulation. Sun *et al*.'s^[37]^ study focused on the phenotype (including morphology and immunophenotype), karyotype, cytokine expression, and hematopoietic support of BM-MSCs in SLE patients.^[38]^ They found that MSCs derived from bone marrow in SLE showed evidence of growth retardation in vitro, and decreased secretion of some cytokines, but had a normal karyotype and supported hematopoiesis. The BM-MSCs from healthy controls could be cultured successfully, and they were still active when cultured for 40 passages, while SLE BM-MSCs grew slowly, and they showed signs of aging when expanded to the 10th generation. The phenotype of SLE BM-MSCs is normal, which is CD29^+^, CD44^+^, CD105^+^, CD14^−^, CD34^−^, CD45^−^, and HLA-DR (human leukocyte antigen DR)^−^. However, the secretion of transforming growth factor β (TGF-β1) and IL-6 and IL-7 mRNA in SLE BM-MSCs were decreased, which may cause hematopoietic damage and immune imbalance.^[37]^ SLE patients’ BM-MSCs were defective in inhibiting T cell and B cell proliferation, as well as plasma cell terminal differentiation.^[39,40]^ It can be hypothesized that such defects lead to immune and hematopoietic dysregulation, resulting in clinical disease. Meanwhile, the ultra-structures of SLE BM-MSCs showed obvious aging characteristics.^[41]^ These abnormalities may be related to the decrease of amplification ability of SLE BM-MSCs in vitro. Activated NF-κB pathway in SLE BM-MSCs inhibited BMP (bone morphogenetic protein)-2–induced osteogenic differentiation through BMP/Smad signaling pathway, which may be involved in the pathogenesis of SLE osteoporosis.^[38]^ In an SLE animal model, Wang *et al*. found that MSCs from NZB/W F1 mice or BALB/c mice could reduce CD3^+^T cells apoptosis; moreover, when co-cultured with T lymphocytes, MSCs from NZB/W F1 mice increased Th2 subsets, while MSCs from BALB/c mice increased Th1 subsets.^[39]^ El-Badri *et al*.^[42]^ found that the immunosuppressive function of MSCs from autoimmune mice was weaker than that of normal MSCs.

### MSC transplant (MSCT) in SLE

All the studies above confirmed that SLE MSCs were abnormal and involved in the occurrence and development of lupus diseases. Hence, the MSCT was used in the treatment for SLE. In 2007, for the first time, Sun *et al*.'s^[43]^ research team began to carry out the allogeneic BM-MSCs transplantation for the treatment of refractory active SLE patients. Four CTX/glucocorticoid treatment-refractory SLE patients using allogeneic MSCT and showed a stable 12–18 months disease remission in all treated patients: that disease activity was satisfactorily controlled, proteinuria and serum autoimmune antibodies declined. In addition, the proportion of CD4^+^ Foxp3^+^ Treg in patients’ peripheral blood increased significantly. This represents the first occasion on which it is proved that allogeneic BM-MSCs transplantation is safe and effective in the treatment of refractory SLE. Subsequently, Sun *et al*.^[44]^ reported the phase I clinical study of allogeneic BM-MSCs and UC-MSCs transplantation in the treatment of refractory SLE.^[45]^ The results showed that transfusion of allogeneic BM-MSCs and UC-MSCs significantly resulted in disease remission, decreased proteinuria, improved renal function, increased serum albumin and complement level, and decreased level of autoantibody in patients with severe lupus, who were otherwise poorly responsive to conventional therapy. Meanwhile, the ratio of Treg cells in patients’ peripheral blood increased significantly after transplantation, accompanied by the increase of TGF-β and the decrease of IL-10.^[44,45]^

A further phase II study enrolled 87 patients with refractory SLE, with up to 4 years of follow-up (mean: 27 months). Of the patients, 59% (51/87) were pretreated with CTX before transplantation, 41% (36/87) patients were not pretreated with CTX, 69 patients were treated with MSCs once (23 patients were treated with BM-MSCs and 46 patients were treated with UC-MSCs), and 18 patients were treated with MSCs multiple times because of their disease condition. This study demonstrated a good clinical safety profile, with an overall rate of survival of 94%, and about 50% patients achieving and remaining in clinical remission at 4 years’ visit, although relapses of disease occurred in 23%.^[46]^ It was found that serum albumin and complement C3 levels increased significantly after transplantation, while SLEDAI score, 24-h proteinuria, serum creatinine and BUN (blood urea nitrogen), ANA and anti-ds DNA antibody decreased significantly. For patients with hematologic system involvement, hemoglobin and platelet levels increased significantly after MSCT. These studies also showed no difference in clinical efficacy between allogeneic bone marrow and UC-derived MSCT. Moreover, there is no significant difference demonstrated between CTX pretreatment group and non-CTX pretreatment group. After transplantation, the patients’ condition was stable and the dosage of glucocorticoid and immunosuppressant decreased. MSCs infusion induced remission in multi-organ dysfunctions including lupus nephritis,^[47]^ diffuse alveolar hemorrhage,^[48]^ and refractory cytopenia.^[49]^

Next, Wang *et al*.^[50]^ conducted a multicenter clinical study, which recruited 40 patients with active SLE, from 4 different clinical centers in China. Each patient was given MSCs intravenous infusion twice, with a week's interval and a dose of 10^6^/kg body weight. After 12 months of follow-up, 6 cases of non-transplant–related adverse events were found (3 patients died and 3 patients developed infection events). The overall survival rate was 92.5%, while the clinical response rate was 60% (32.5% patients reached major clinical response; 27.5% patients achieved partial clinical response). After transplantation, the scores of SLEDAI and BILAG decreased significantly. The levels of serum albumin and complement C3 increased significantly; 24-h proteinuria, serum creatinine, and BUN decreased significantly. Meanwhile, MSCT could improve BILAG score of blood system and skin system. Again, a proportion of patients (17.5%) experienced disease relapse within 6 months of a prior clinical response and required repeated MSCT (Table 1).

**Table 1:** Summary of clinical trials of MSCs in SLE patients

**Recipient**	**Cell source**	**Patients No.**	**Sex**	**Graft type**	**Results**	**Study type**	**Reference**
SLE patients with LN	Human BM Human UC	81	74 (F), 7 (M)	1 × 10^6^ cells/kg intravenous	Renal remission, represented by decreased proteinuria and serum creatinine levels. Improved SLEDAI score, GFR, increased total serum albumin and decreased anti-ds DNA antibody level.	Open-label clinical trial	[047]
SLE patients with refractory cytopenia	Human BM	35	34 (F), 1 (M)	1 × 10^6^ cells/kg intravenous	SLEDAI decreased; improved leukopenia, thrombocytopenia and anemia; increased Treg/Th17 ratio.	Retrospective study	[049]
SLE patients	Human BM	15	14 (F), 1 (M)	1×10^6^ cells/kg intravenous	Decreased SLEDAI, anti-ds DNA, ANA, proteinuria, creatinine, and BUN; improved skin complications, arthritis, refractory hypertension; increased Treg.	Phase I clinicalstudy	[045]
Diffuse alveolar hemorrhagic SLE patients	Human UC	4	4(F)	1 × 10^6^ cells/kg intravenous	Improved hemoptysis, dyspnea, hypertension, gingival and vaginal bleeding. Amelioration of anemia, thrombocytopenia and serum albumin. Decreased immune cell infiltration and DAH.	Retrospective study	[048]
MRL/lpr mice SLE patients	C3H/HeJ BM Human BM	4	3 (F), 1 (M)	≥1 × 10^6^ cells/kg intravenous	Improved kidney and liver function, increased bone and marrow generation, increased HSC niche creation, increased Treg/Th17 ratio.	Animal study	[042]
SLE patients	Human UC	16	14 (F), 2 (M)	1 × 10^6^ cells/kg intravenous	Decreased SLEDAI, 24-h proteinuria, BUN, serum albumin, anti-ds DNA and ANA, increased C3 level.	Phase I clinicalstudy	[044]
SLE patients	Human UC Human BM	81	76(F), 5(M)	1 × 10^6^ cells/kg intravenous	Follow-up 5 years following experiment: decreased SLEDAI, proteinuria increased serum albumin, C3 level, white blood cell count and hemoglobin.	Long-term follow-up study	[053]
SLE patients	Human UC	40	38 (F), 2 (M)	1 × 10^6^ cells/kg intravenous	Decreased disease activity, anti-ds DNA, antinuclear antibody, serum albumin, complement proteins, proteinuria, BUN and serum creatinine levels.	Multicenter clinical study	[050]
SLE patients	Human UC Human BM	87	80 (F), 7 (M)		Follow-up 4 years following experiment: improved SELENA-SLEDAI scores, decreased 24-h proteinuria, serum creatinine, BUN, and GFR. Improved hemoglobin and platelet counts became normal.	Phase II clinicalstudy	[046]

HSC, Hematopoietic stem cell; MSCs, mesenchymal stem cells; SLE, systemic lupus erythematosus; SLEDAI, systemic lupus erythematosus disease activity index.

What is the appropriate dose of infused MSCs? Wang *et al*.^[51]^ further compared the clinical efficacy between single and double infusions of MSCs in lupus patients. Of the patients, 58 with refractory SLE were enrolled in the study: 30 in single infusion group and 28 in double infusion group. The average follow-up period was 26–27 months. The results showed that the survival rates of the 2 groups were 100% and 96.4%, and the clinical complete remission rates were 53.3% and 28.6%, respectively. The recurrence rates were 26.7% and 22.2%, respectively. In the 2 groups, 86.7% and 88.9% of the patients had renal involvement, respectively. 24-h proteinuria decreased significantly after MSCT, especially in the single infusion group at 12-month follow-up. The level of serum creatinine of patients with renal insufficiency decreased significantly after transplantation, no matter in which group. Of the patients, 23.3% in single infusion group and 32.1% of the patients in double infusion group had infection-related adverse events, but there was no significant difference between the 2 groups. At the last follow-up, 2 patients in both groups stopped using immunosuppressant; 80.0% of patients in single infusion group and 78.6% in double infusion group used maintenance dose of glucocorticoid 5–10 mg/d.

Several surveys were conducted to estimate the long-time safety of MSCT for patients with refractory SLE, since the safety profile should be strictly concerned. Wang *et al*.^[52]^ enrolled 9 refractory SLE patients, and all the patients completed MSCs infusions twice. One patient had mild dizzy and warm sensation 5 min after MSCs infusion, and the symptoms disappeared quickly. No other adverse event was observed. There was no change in peripheral white blood cell count, red blood cell count, and platelet number in these patients after 6 years’ follow-up. Liver functional analysis showed that serum alanine aminotransferase, glutamic-oxalacetic transaminase, total bilirubin, and direct bilirubin remained in normal range after MSCs infusions. No newly onset abnormality was detected on electrocardiogram and chest radiography. Moreover, there was no rise of serum tumor markers, including AFP (alpha fetoprotein), CEA (carcinoembryonic antigen), CA125, and CA199, before and 6 years after MSCs infusions. The long-term observational study demonstrated a good safety profile of allogeneic UC-MSCs in SLE patients.

### Comparison Between HSCT and MSCT

The experience of patients with SLE treated by HSCs transplantation is reviewed and summarized: the curative effect is positive, but the incidence of adverse reactions is high, as well as the expense and the recurrence rate. Therefore, its clinical application is greatly limited. However, since 2007, Sun's team for the first time successfully carried out allogeneic BM-MSCT in the treatment of refractory SLE.^[37]^ In the clinical study of patients with SLE, the long-term follow-up effective rate is 60%, and there is no obvious adverse reaction. The comparison between HSCT and MSCT is shown in Table 2.

**Table 2:** Comparison between HSCT and MSCT for SLE

	**HSCT**	**MSCT**
Therapeutic effect	Some SLE patients achieve remission, however, relapse rate is nearly one-third.^[23]^	More than half of SLE patients exhibited complete and partial clinical remission after MSCT.^[47,52]^ And MSCT induce remission in multi-organ dysfunctions including lupus nephritis.^[48]^
Therapeutic mechanism	HSC itself seems to have no direct therapeutic effect; it depends on several other mechanisms: (1) High-dose immunosuppression during HSCT would eliminate autoreactive lymphocytes, and the development and re-organization of a self-tolerant immune system after HSCT would be effective.^[32]^ (2) The level of regulatory T cell numbers changes, and the pathogenic T cell responses against autoantigens are inhibited.^[55]^	The therapeutic effect of allogeneic MSCT is primarily dependent on the systemic immunoregulatory effects on various immune regulatory cells, including T cells,^[39]^ B cells,^[40]^ plasma cells,^[55]^ dendritic cells,^[56]^ macrophages,^[57]^ *et al*. MSCs also secrete a variety of anti-inflammatory cytokines that mediate immune response. Also, MSCs could be home to kidney, lung, liver, and spleen tissues and may contribute to regulate local inflammation.^[58,59]^
Adverse events	Infection (CMV or bacterial/fungal),^[20]^ secondary AD,^[35]^ allergy, elevation of liver enzymes, bone pain, and heart failure, *et al*.^[34]^.	Only a small number of patients have mild side effects such as dizzy and warm sensation.^[48]^
Cost	Relatively high expense for complicated cell conditioning and high-dose immunosuppressive drugs	MSCs have strong tissue repair function and multi-directional differentiation potential such as HSCs, and exhibit low immunogenicity and immunosuppressive ability. It has advantages in terms of patient acceptance and the cost is much lower than that of HSCs.

AD, autoimmune diseases; HSC, Hematopoietic stem cell; HSCT, HSCs transplant; MSCs, mesenchymal stem cells; MSCT, MSC transplant; SLE, systemic lupus erythematosus.

In recent years, MSCs therapy is rapidly developing, and they have an important clinical application prospect in the treatment of many AD, especially in the treatment of SLE. There are many abnormalities in the structure and function of bone marrow MSCs in SLE, suggesting the necessity for allogeneic MSCs transplantation. Nowadays, a large number of animal experiments have confirmed the efficacy of allogeneic MSCs transplantation in the treatment of lupus mice. Phase I and phase II clinical studies have confirmed the safety and efficacy of allogeneic MSCs in the treatment of SLE patients. Further results from large clinical trials are needed to confirm preclinical findings, and at the same time fully and profoundly clarify the mechanism of MSCs treatment.
